# Richmond Academy of Medicine and Surgery

**Published:** 1901-08

**Authors:** 


### SOCIETY REPORTS.

### RICHMOND ACADEMY OF MEDICINE AND SURGERY.

Regular meeting held June 25, 1901, Stuart McGuire, M.D., President, in the chair; Mark W. Peyser, M.D., Reporter.

*Dr. A. L. Gray* read a paper on "Tonsillitis from the Standpoint of the General Practitioner." (See paper on page 312.)

DISCUSSION.

*Dr. Jacob Michaux* agreed that a specific cause would probably be found for tonsillitis. The comparatively high temperature accompanying such a limited focus of infection seemed to argue a particularly virulent germ. He had obtained better results from the simple tincture of guaiacum, in small doses frequently repeated, than from the ammoniated tincture. Regarding tonsillar pus formation, he could not recall a collection existing in the gland itself. He had found some difficulty in keeping open the incision, especially in children.

*Dr. J. S. Wellford* disagreed as to the infectiousness of tonsillitis, for he had seen numbers of individuals who had several attacks, other members of the family escaping. In elderly people, the tonsils were often absent, due to destructive effects of abscesses; and he, therefore, was of the opinion that these abscesses were tonsillar and not peritonsillar, the pillars being still present. He thought that follicular tonsillitis in the early stage, was easily diagnosed from diphtheria, the latter almost invariably commencing at one point and radiating therefrom, while the former involved a number of points at first separate, but later coalescing. In nine out of ten cases, thorough cauterization of each point with nitrate of silver and the use of an astringent gargle were all that was necessary.

*Dr. H. H. Levy* said that follicular tonsillitis could be invariably diagnosed if seen before thirty-six hours had elapsed from the beginning of invasion, because of the isolated spots which represented exudation in the lacunæ. Notwithstanding Jacobi's statement, he had never seen diphtheria begin in spots, and to disprove it had had made often bacteriological examinations. The disease could be cut short by proper treatment. An ordinary case recovered of itself in five days, sometimes three days, if the throat were kept clean, as by a salt wash. Where there was headache, intense limb pain and high temperature, he gave sodium salicylate. If the temperature were not high and pain not great, he gave internally, bichloride of mercury and tincture of iron; and he had seen a spray composed of bichloride of mercury, gr. j, chloride of ammonium, gr. xx and water, ℥ij, used every hour or two, cause entire disappearance of the deposit in thirty-six hours.

*Dr. R. D. Garcin* did not believe in the bacterial origin of tonsillitis, his experience coinciding with that of Dr. Wellford. He did believe, however, that rheumatism was a predisposing cause. In his own case, salicylate of sodium was the only agent that gave relief; and in another in which there was a temperature of 107 degree F., and the tonsils had been twice lanced, the remedy reduced it. His patients did not recover in five days, though the acute symptoms might subside in that time.

*Dr. Levy* said that he and most physicians believed that bacteria did cause disease; that a great many of them existed in the mouth; and that physicians ought to preach that under certain circumstances they might cause disease in various parts of the body. In a number of hospitals, cleansing the mouth was preliminary to operation, because during anesthesia the patient might suck material into the lungs and produce pneumonia. The absolute strength of the body depended upon the health of the cells of the body; and that part which was weak fell prey, under exposure, to disease; that healthy did not.

Regarding its duration, he had never seen follicular tonsillitis last longer than five days.

*Dr. Wellford* rejoined that it was not necessary to explain inhalation pneumonia by a germ which might have remained dormant otherwise. Ether was a compound body the decomposition of which to obtain its oxygen strained the lungs. Chloroform did not produce the disease because it did not contain oxygen.

He contended that nine-tenths of all affections were due to ptomaines, not bacteria. If the cells were weak why could they not produce disease in the absence of germs?

*Dr. W. F. Mercer* stated that pus in the tonsils was rare. He had seen but one or two cases in which it had originated as the follicular form. In differentiating tonsillitis from diphtheria, it would be well to bear in mind that the exudate was more of a whitish-yellow and that in every case the tongue was heavily coated, which, as a rule, was not true of diphtheria at first.

*Dr. Gray*, in closing the discussion, said that he had often noticed the discrete follicles mentioned by Dr. Wellford, and thought them a valuable point; but had never seen account of them as of diagnostic value in any literature. As to the infectiousness of the disease, he had read an article since writing his paper, which stated that follicular tonsillitis was an acute infection having an incubation period of four days. He thought it strongly probable that this was true, but whether this was due to a germ existent in the throat in health, or one adventitious, no one could say. He believed that some individuals were predisposed to it; that by some hygienic violation, a point of diminished resistance was produced in the tonsils; but without the added germ, the disease was impossible. He agreed with Dr. Levy regarding this. He had found that ninety per cent. of his cases of follicular tonsillitis never had rheumatism; but it would be strange if two such common affections were not coincident at some time.

---

AMERICAN MEDICINE is authority for the following: A surgeon-general of the German army is reported to have treated tobacco leaves with tannin and a decoction of origanum vulgare before they were made into cigars, and by this means so transformed the contained nicotine as to render it harmless to the human system. Experiments were made upon persons having aversion to tobacco in any form, and these smoked three cigars in succession without experiencing any inconvenience, withoutalteration in pulse, breathing, or temperature.

